# About Dental Colleges

**Published:** 1892-07

**Authors:** 


### EDITORIAL.

### About Dental Colleges.

There is, perhaps, no subject of greater interest to the progressive dentists of our country than that of the education of those who propose to enter its ranks in the future. The method of the education of dentists is very different from that of comparatively a few years ago. About fifty years ago the only method of attaining a knowledge of dentistry was by means of private pupilage. This, in some instances, when directed by and under the supervision of preceptors who realized their responsibility, brought forth good results. These, however, were few compared with the whole. The rule was that the education of the dentist, till within comparatively a few years, was a very meagre affair indeed, and pertained only to practical matters; the science of the profession was wholly passed over. Now, however, the matter is wholly changed—the rule is a special college training, as a preparation for entering upon dental practice. This change has increased the demand for educational facilities, and on that account, in part at least, the number of dental colleges has been rapidly increasing for the last few years; and in that matter there seems to be, as yet, no dropping off in organizing colleges. Anything like a thorough knowledge of college work and of what is being done in an educational way is very manifest to one who thinks upon the subject. Information is usually obtainable through the annual announcements of the various colleges as to their future work, but to these not much attention is usually given, except, perhaps, by those who propose to be students in the various colleges, and they are more concerned about the future than the past.

While thinking upon this subject somewhat, we have decided to refer to a few of the colleges; perhaps, we may speak of others in the future. First, of the American College, of Chicago.

This institution, in some respects, has had a prosperous career during the last six years, especially in reference to the number of students in attendance. In some particulars of its management, however, its course was regarded as deviating widely from the requirements established by the National Association of Dental Faculties, and upon a pretty thorough investigation held at the last meeting of this body, the American College was suspended from membership in that organization. That action also placed it under the ban of the National Association of Boards of Dental Examiners. This constitutes an embarrassment that no college can afford to stand beneath. This action placed it in a very unfavorable light before the profession, and as a result its classes were greatly reduced during the last year. So serious did the matter seem to the management, that they were willing for any change that would relieve them of the difficulty, and, if possible, put the institution upon a more favorable basis. With a view to this consummation the entire management has been changed, those who have had charge of its affairs have disposed of their interest and withdrawn; a new faculty has been organized and a different management inaugurated.

The faculty now consists of Dr. John S. Marshall, M.D., Dean; Drs. L. C. Ingersoll, R. F. Ludwig, E. L. Clifford, I. B. Crissman, B. J. Cigrand, Vida A. Latham, Thomas G. Rix, H. B. Harrison, Weller Van Hook, Geo. Leininger, T. B. Wiggin, W. M. Tanquary, and H. D. Coghlan.

In addition to these there is a full corps of clinical demonstrators. The announcement states "that the financial management of the College has passed under the control of a syndicate of business and professional gentlemen, whose aim will be to make this institution one of the foremost in the land." There is evidently room for this institution, and the promise now seems to be, under the changed condition, that good work will be done by it.

Ohio seems to be coming to the front, during the last year and a half, in the establishment of dental colleges. Ohio had the first dental college in the West, and the second one in the world, and the wonder is, with her restless, progressive dental profession that she has rested so long with only one college, while some of her sister states have charters for fifteen or more dental colleges, and eight or ten in actual operation.

The first pronounced movement for the second dental college in Ohio was made about two years ago, in connection with the Homœopathic Hospital College, of Cleveland, O. That organization was effected a year ago, with Dr. W. H. Whitslar as Dean and Professor of Principles and Practice of Dental Science; Drs. Geo. H. Wilson, H. F. Harvey, L. P. Bethel, J. E. Robinson, S. B. Dewey, and I. E. Sampsell as teachers of the various branches. A regular session was held during the past year which gave a good degree of encouragement for the future. There were present fifteen matriculates; there were six graduates.

Some change has recently been made in the faculty by the resignation of Dr. W. H. Whitslar and Dr. Geo. H. Wilson. These vacancies have been filled, and preparations are being made for a still larger class during the coming year.

The withdrawal of Drs. Whitslar and Wilson from the college just referred to was that they might enter into a new organization which has, within the last three or four months, been effected. This is the Dental Department of the Western Reserve University, in Cleveland, Ohio. This is so far effected that the annual announcement for the session of '92-'93 has been issued. By that we see the special faculty consists of Charles F. Thwing, D.D.. President; C. R. Butler, Dean, Dr. W. H, Whitslar, Secretary, Drs. Geo. H. Wilson, H. F. Harvey, D. R. Jennings, J. R. Owens, H. L. Ambler, and J. W. Van Doorn. This department is organized in connection with a very prosperous institution, one that has been well established for many years. The aim, as stated in the announcement, is to make a high standard of requirements in all particulars; and judging by the men who constitute the faculty there is scarcely a doubt as to the execution of the plan to the full.

In addition to this a new Dental Department is being organized at Columbus, O., in connection with a new medical school —The Ohio University Medical College. This is an institution, as we have been informed, with large resources, and will be able to plan and carry out large ideas. The dental department is being organized concurrently with the medical. Dr. A. F. Emminger is a member of the Board of Trustees of the medical college, and has placed upon him the responsibility of organizing the dental faculty—by no means a minor matter. Dr. Emminger has already been assigned to the position of Dean of the Dental Faculty. Buildings are being erected for the use of the medical and dental departments; they will have the latest and most approved construction, and be adapted especially for the various lines of work to be done.

The dental department of the Cincinnati College of Medicine and Surgery, Cincinnati, O., was organized last year. It, like the others, is a department of a medical college. It held a session during the last year with thirty-four matriculates, and at the recent commencement had ten graduates. The special faculty consists of Drs. G. S. Junkerman, Dean; John M. Shaller, W. E. Lewis, A. I. F. Buxbaum, and W. T. McLean. In the announcement it is said "that the aim of this school is to teach every department of dental surgery to completion." It is also said, "that this department will conform to all the rules and regulations, and all the requirements governing the National Association of Dental Faculties." It has a six months' session; three full courses are required for the degree of D.D.S. Indeed, the promise is on the part of all dental colleges recently organized, and those being organized, that they will conform fully to the requirements of the National Association of Dental Faculties, and we trust this will be done in good faith; and even if some or all of them should raise their standards higher than this it would be to their praise.

From a circular just at hand, we see that a dental department has been organized in connection with, and under the auspices of the University of Buffalo, New York. The organization is about completed, and a session will be held the coming Winter. This, like most new colleges of the day, proposes to take a high stand, and make very thorough work in its course of instruction. The principal men of the dental faculty are: Drs. W. C. Barrett, A. P. Southwick, Secretary; F. E. Howard, Herbert A. Birdsall, and J. Edward Line. Additional teachers will be added as may be required. This department will have ample accommodation, and all facilities for its work; this is one of the advantages of dental colleges being allied with other and large institutions.

The organization of the department has occasioned some excitement and friction in the profession in Buffalo, the merits of which we do not care to discuss here, feeling well assured that if not already, there will be enough discussion upon the subject with the profession in Buffalo and vicinity. It is always better to keep a family disturbance within a household, as little good is ever accomplished by turning it loose upon a community; so, all we have to say in reference to that is—" Gentlemen, settle your little difficulty at home, you have nothing to gain by trying to get somebody else to do it."

Judging from the character of those engaged in this enterprise and from what is known of the University of Buffalo, we doubt not, this department will make for itself a good record.

